# Expression level of the reprogramming factor NeuroD1 is critical for neuronal conversion efficiency from different cell types

**DOI:** 10.1038/s41598-022-22802-z

**Published:** 2022-10-26

**Authors:** Kanae Matsuda-Ito, Taito Matsuda, Kinichi Nakashima

**Affiliations:** Stem Cell Biology and Medicine, Department of Stem Cell Biology and Medicine, Graduate School of Medical Sciences, Kyushu University, 3-1-1, Maidashi, Higashi-Ku, Fukuoka, 812-8582 Japan

**Keywords:** Cell biology, Neuroscience, Stem cells

## Abstract

Several transcription factors, including NeuroD1, have been shown to act as neuronal reprogramming factors (RFs) that induce neuronal conversion from somatic cells. However, it remains unexplored whether expression levels of RFs in the original cells affect reprogramming efficiency. Here, we show that the neuronal reprogramming efficiency from two distinct glial cell types, microglia and astrocytes, is substantially dependent on the expression level of NeuroD1: low expression failed to induce neuronal reprogramming, whereas elevated NeuroD1 expression dramatically improved reprogramming efficiency in both cell types. Moreover, even under conditions where NeuroD1 expression was too low to induce effective conversion by itself, combined expression of three RFs (Ascl1, Brn2, and NeuroD1) facilitated the breaking down of cellular barriers, inducing neuronal reprogramming. Thus, our results suggest that a sufficiently high expression level of RFs, or alternatively their combinatorial expression, is the key to achieving efficient neuronal reprogramming from different cells.

## Introduction

Lineage-specific transcription factors induce direct reprogramming of somatic cells to other cell types, such as neurons, without transit through a pluripotent stem cell state. In 2010, mouse fibroblasts were shown to be directly converted to neurons in vitro by forced expression of three transcription factors, Ascl1, Brn2, and Myt1l^1^. More recently, by examining combinations of factors used for reprogramming, it has become possible to convert fibroblasts to neurons of specific subtypes, such as sensory and motor neurons^2,3^. Several groups have reported in vivo neuronal reprogramming into neurons from glial cells, including astrocytes, which become reactive after brain damage and contribute to eventual glial scar formation. For example, ectopic expression of Sox2, Neurog2, or NeuroD1 converts endogenous astrocytes to neurons in the mouse brain^4–7^. Microglia, another type of glial cell, are the resident immune cells in the brain and are derived from primitive macrophages^8^. Microglia accumulate at the injured site to remove dead cells after brain injury, such as stroke, and consequently become the predominant cell type in the ischemic core region^9^. We have previously shown that microglia can be converted into neurons both in vitro and in vivo by the ectopic expression of lentivirus-encoded NeuroD1^10^. Although in vivo neuronal reprogramming from these two glial cells holds great promise as a therapeutic strategy, further improvement of neuronal reprogramming efficiency is desirable to supply sufficient numbers of new neurons for complete functional recovery from neurological injury and diseases.

In a previous report, single-cell RNA sequencing analysis indicated that high but not low expression of exogenous Ascl1 induced the expression of neuronal marker genes in fibroblasts^11^, implying a correlation between transgene expression level and the attainment of neuronal reprogramming. However, it has not been extensively studied how the conditions in which reprogramming factors (RFs) are expressed influence neuronal reprogramming efficiency. Here, we examined neuronal reprogramming from microglia and astrocytes under conditions of different expression levels of NeuroD1 in these two glial cell types. In contrast to the higher expression, when we decreased the expression of NeuroD1 by reducing doxycycline (Dox) concentration, the neuronal conversion from microglia was dramatically diminished. On the other hand, increasing the NeuroD1 expression level by repeating the lentiviral infection (i.e., 2 times) improved neuronal reprogramming efficiency from microglia. Moreover, multiple NeuroD1-expressing viral infections (3 times) enabled neuronal reprogramming from non-reactive (NR-) astrocytes that were previously shown to be resistant to conversion into neurons with a single infection^10^. We also found that the combined expression of three RFs, Ascl1 and Brn2 together with NeuroD1, efficiently induced neuronal reprogramming, even when their expression was low. Taking these observations together, we believe that our study offers efficient strategies to reprogram neurons from glial cells and will contribute to accelerating the development of therapeutic applications for brain injury and diseases.

## Results

### NeuroD1 expression level-dependent changes in reprogramming efficiency

**Figure 1:**
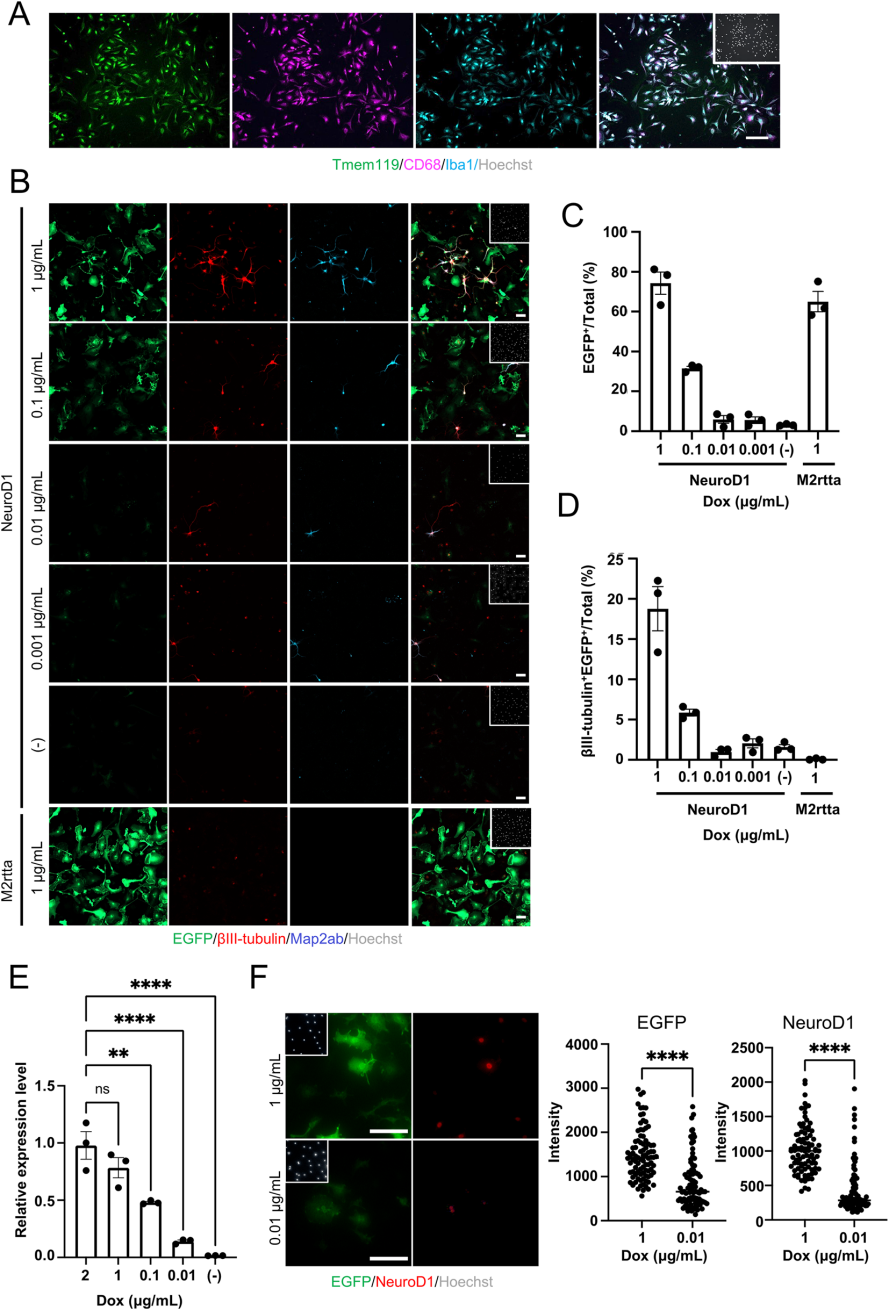
Surpassing an RF expression threshold is required to convert microglia efficiently into neurons (**A**) Representative images of staining for microglial markers Tmem119 (green), CD68 (magenta), and Iba1 (cyan) in primary isolated microglia. Scale bar, 75 μm. (**B**) Representative images of staining for EGFP (green), and the neuronal markers βIII-tubulin (red) and Map2ab (cyan), at 7 days post-transduction (dpt) of microglia infected with three viruses (EGFP-, M2rtta-, and NeuroD1-viruses; indicated as NeuroD1) or two viruses (EGFP- and M2rtta-viruses; indicated as M2rtta) under the indicated Dox concentration treatment conditions. Scale bars, 50 μm. (**C**) Quantification of the EGFP^+^ cells in (**B**) (n = 3). (**D**) Quantification of the βIII-tubulin^+^ EGFP^+^ cells in (**B**) (n = 3). (**E**) qRT-PCR analysis of total *NeuroD1* mRNA levels in NeuroD1-transduced microglia at 2 dpt under the indicated Dox concentration treatment conditions (n = 3 biological replicates). ^**^p < 0.005, ^***^p < 0.0005, ^****^p < 0.0001 by ANOVA with Tukey post hoc tests. ns means not significant (*p* > 0.05). (**F**) Representative images of staining for EGFP (green) and NeuroD1 (red) in NeuroD1-transduced microglia at 2 dpt under 1 or 0.01 μg/mL Dox induction (left). Intensity of EGFP or NeuroD1 in left panel (right). ^****^p < 0.0001 by unpaired Student’s t test. ns means not significant (*p* > 0.05). Scale bars, 75 μm.

To investigate whether the efficiency of microglia-to-neuron (MtN) reprogramming is influenced by the expression level of NeuroD1, we used a lentivirus expressing NeuroD1 under the control of the Dox-inducible tetracycline response element promoter (NeuroD1-virus). We obtained highly enriched Tmem119-, CD68-, and Iba1- positive (Tmem119^+^ CD68^+^ Iba1^+^) microglia from the 1-day-old mouse cortex as in our previous study^10^ (Fig. 1A) and added an equal amount of individual viruses to each microglial culture dish, but treated them with different doses of Dox. We observed the Dox dose-dependent appearance of EGFP^+^ cells in EGFP-, M2rtta-, and NeuroD1-virus-infected cells (Fig. 1B,C). We also confirmed that almost no βIII-tubulin^+^ cells emerged in the microglia infected with EGFP- and M2rtta- but without NeuroD1-virus even when treated with 1 µg/ml Dox (Fig. 1B and D), indicating that there were very few, if any, contaminating neurons and/or cells with spontaneous neurogenic potential in the isolated microglial population. We then scrutinized the neuronal conversion efficiency of EGFP- and M2rtta- together with NeuroD1-virus-infected microglia in the individual dishes based on the proportion of βIII-tubulin^+^/EGFP^+^ cells among Hoechst^+^ total cells and found that the efficiency was greatly decreased by reducing the Dox concentration (Fig. 1D). Consistent with this, although no further increase of NeuroD1 expression was observed at 2 µg/mL of Dox compared to 1 µg/mL (Fig. 1E), the NeuroD1 expression decreased in a Dox concentration-dependent manner (Fig. 1E), suggesting that a low level of NeuroD1 expression cannot effectively induce MtN conversion but that NeuroD1 per se is able to do so if highly expressed.

We have previously reported that the expression levels of exogenous NeuroD1 are highest at 2 days after Dox induction (1 μg/mL) in microglia^10^. However, almost no cells exhibit neuronal morphology at this stage; such cells appeared at 7 days post-transduction (dpt)^10^. We also confirmed that the expression level of exogenous *NeuroD1* mRNA is highest at 2 days after 0.1 and 0.01 μg/mL Dox induction (Supplemental Fig. 1A) as in the case of 1 μg/mL Dox treatment. Moreover, the proportions of NeuroD1-converted βIII-tubulin^+^EGFP^+^ cells from microglia were not higher at 10 days after both 1 and 0.01 μg/mL Dox induction than those at 7 days (Supplemental Fig. 1B,C), suggesting that ratios of EGFP^+^ or βIII-tubulin^+^ cells in total cells reached peaks at 7 dpt when exogenous NeuroD1 expression had been downregulated.

Next, to compare the protein expression levels of NeuroD1 per cell under different Dox concentrations, we treated virus-infected microglia with Dox at 1 or 0.01 µg/mL, and observed reduced protein expression of both NeuroD1 and EGFP at single-cell resolution at the lower dose relative to the higher one (Fig. 1F). These data indicate that NeuroD1 expression above a certain threshold level is required for the effective induction of MtN conversion.

### Elevated expression level of NeuroD1 enhances neuronal reprogramming

**Figure 2:**
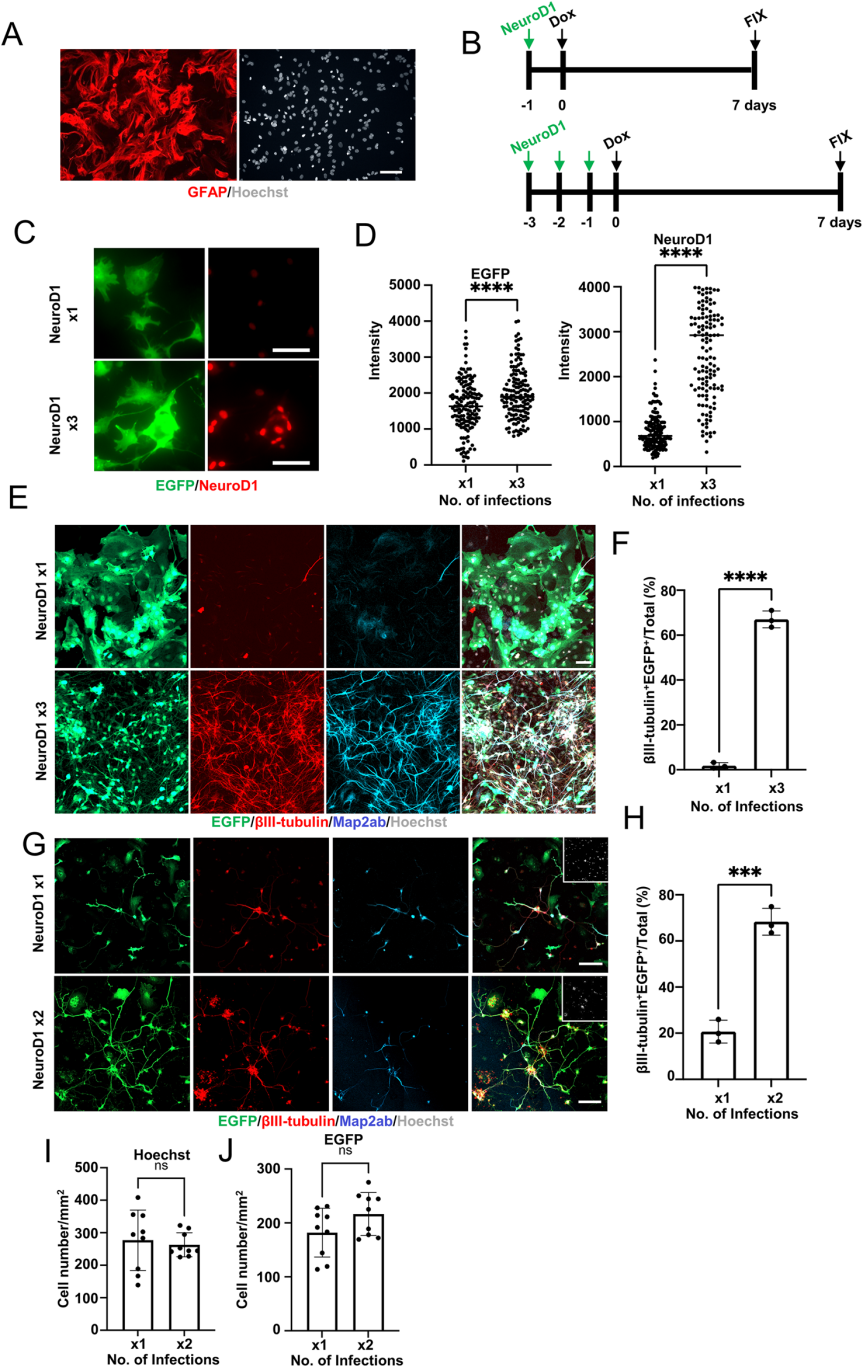
Conversion efficiency is promoted with increased RF expression level. (**A**) Representative images of non-reactive astrocytes (NR-astrocytes) stained for the astrocyte marker GFAP (red). Scale bar, 75 μm. (**B**) Scheme of single or multiple (three times) infection of NR-astrocytes with three viruses (EGFP-, M2rtta-, and NeuroD1-viruses; indicated as NeuroD1). (**C**) Representative images of staining for EGFP (green) and NeuroD1 (red) in NeuroD1-transduced NR-astrocytes at 2 dpt. Dox, 1 μg/mL. Scale bars, 75 μm. × 1 and × 3 indicate number of viral infections. (**D**) Fluorescence intensity of EGFP or NeuroD1 in (**C**). ^****^p < 0.0001 by unpaired Student’s t test. (**E**) Representative images of staining for EGFP (green), βIII-tubulin (red), and Map2ab (cyan) in reprogrammed neuronal cells from NR-astrocytes at 7 dpt. Dox, 1 μg/mL Dox. Scale bars, 100 μm. × 1 and × 3 indicate number of viral infections. (**F**) Quantification of the βIII-tubulin and EGFP^+^ cells in (**D**) (n = 3). ^****^p < 0.0001 by unpaired Student’s t test. (**G**) Representative images of staining for EGFP (green), βIII-tubulin (red), and Map2ab (cyan) in reprogrammed neuronal cells from microglia at 7 dpt. Dox, 1 μg/mL. Scale bars, 100 μm. × 1 and × 2 indicate number of viral infections. (**H**) Quantification of the βIII-tubulin and EGFP ^+^ cells in (F) (n = 3). ^***^p < 0.0005 by unpaired Student’s t test. (**I**) Quantification of the number of Hoechst^+^ cells per mm^2^ in NeuroD1-transduced microglia. × 1 and × 3 indicate number of viral infections. (n = 3). ns means not significant. (**J**) Quantification of the number of EGFP^+^ cells per mm^2^ in NeuroD1-transduced microglia. × 1 and × 3 indicate number of viral infections. (n = 3). ns means not significant.

We next explored whether increasing the expression level of NeuroD1 promotes neuronal reprogramming from astrocytes in addition to microglia. We prepared mouse NR-astrocytes in vitro, treated them with AraC, and allowed them to grow without growth factors; these astrocytes have a state closely resembling that of astrocytes under physiological conditions (Fig. 2A)^11,12^. Since we have previously shown that NR-astrocytes are hardly reprogrammed into neurons by single infection with NeuroD1-virus^10,14^, we attempted to increase the NeuroD1 expression level by performing multiple infections with the viruses in this study. To this end, we first examined whether multiple infections can be achieved in cells by repeated exposures to the viruses: GFAP-expressing NR-astrocytes were exposed first to EGFP-virus and then to tdTomato-virus. As anticipated, we observed EGFP and tdTomato dual-positive cells (Supplemental Fig. 2A,B), indicating that NR-astrocytes were sequentially infected with viruses.

Next, NR-astrocytes were infected once (× 1) or three times (× 3) with the set of the three viruses (EGFP-, M2rtta-, and NeuroD1-viruses) (Fig. 2B), and we found that the repeated infection increased both NeuroD1 and EGFP protein expression levels at the single-cell level (Fig. 2C,D). In addition, elevating the expression of NeuroD1 by the repeated infections dramatically promoted astrocyte-to-neuron (AtN) conversion at 7 dpt compared to the single infection (Fig. 2E,F).

To exclude the possibility that reactivation of astrocytes by repeated virus infections rather than increased NeuroD1 expression promoted AtN conversion, we infected NR-astrocytes twice with viruses without NeuroD1 and subsequently once with the NeuroD1-containing viruses. We observed almost no AtN conversion in this experimental setting, indicating that repeated infection itself does not improve the AtN reprogramming efficiency (Supplemental Fig. 2C,D).

Actually, infecting cells twice (× 2) with the three viruses including NeuroD1 also increased MtN reprogramming efficiency compared to the single (× 1) infection, without further affecting cell survival (Fig. 2G–J).

Taken together, these data indicate that increased NeuroD1 expression enables efficient neuronal reprogramming even from cells that are difficult to convert.

### Differences in cell context affect neuronal reprogramming efficiency

**Figure 3:**
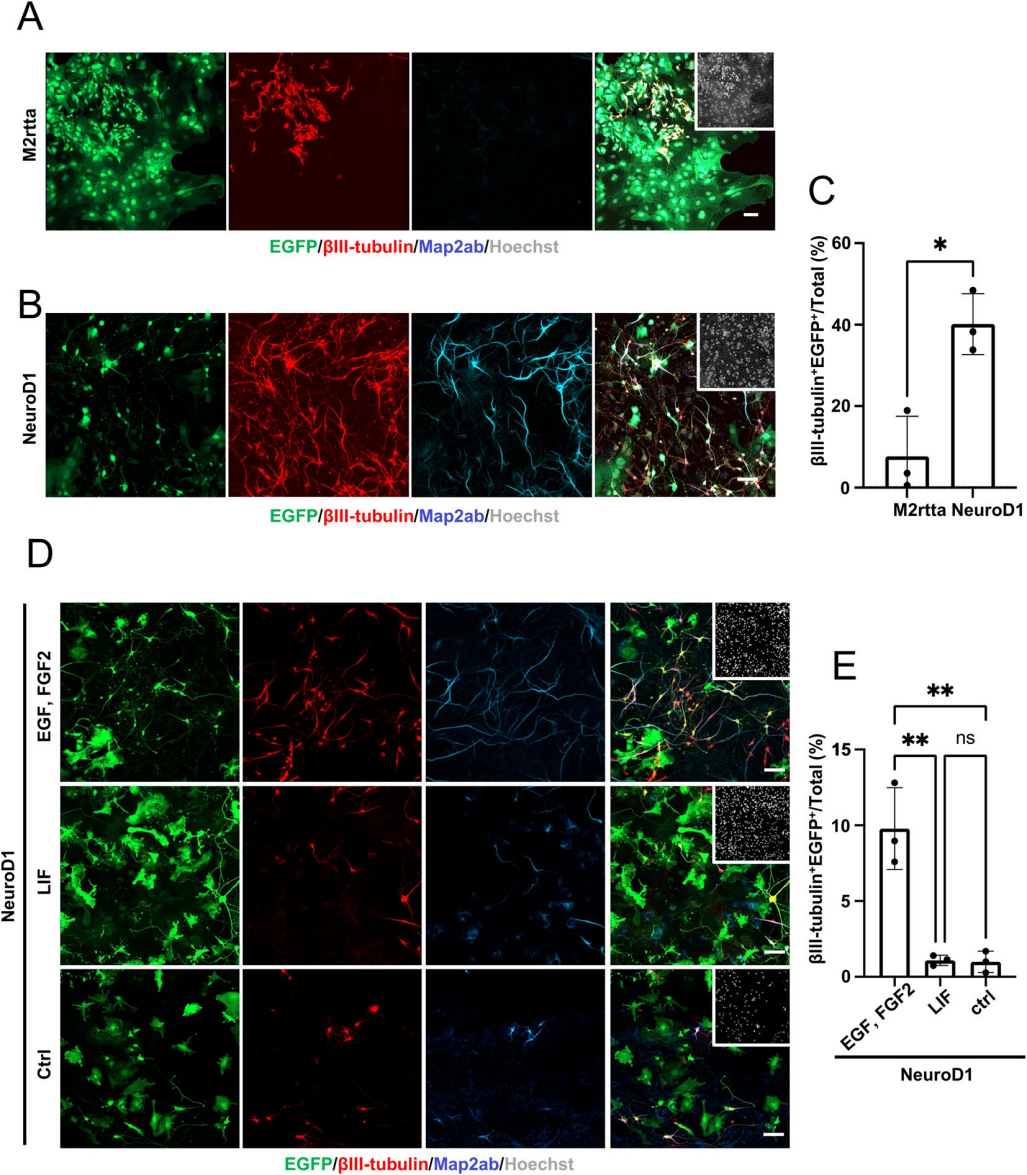
Environmental stimulation affects neuronal reprogramming efficiency. (**A**) Representative images of staining for EGFP (green), βIII-tubulin (red), and Map2ab (cyan) at 7 dpt of astrocytes infected with two viruses (EGFP- and M2rtta-viruses; indicated as M2rtta) and culturing with EGF and FGF2. Dox, 1 μg/mL. Scale bar, 100 μm. (**B**) Representative images of staining for EGFP (green), βIII-tubulin (red), and Map2ab (cyan) at 7 dpt of astrocytes infected with three viruses (EGFP-, M2rtta-, and NeuroD1-viruses; indicated as NeuroD1) and culturing with EGF and FGF2. Dox, 1 μg/mL. Scale bar, 100 μm. (**C**) Quantification of the βIII-tubulin and EGFP ^+^ cells in (**A** and **B**) (n = 3). ^*^p < 0.05 by unpaired Student’s t test. (**D**) Representative images of staining for EGFP (green), βIII-tubulin (red), and Map2ab (cyan) in NeuroD1-transduced NR-astrocytes cultured with EGF and FGF2, LIF, and without these factors (Ctrl) at 7 dpt. EGF and FGF2 or LIF were applied for 3 days. Dox, 1 μg/mL. Scale bars, 100 μm. (**E**) Quantification of the βIII-tubulin and EGFP^+^ cells in (**D**) (n = 3). ^**^p < 0.005 by ANOVA with Tukey post hoc tests. ns means not significant (*p* > 0.05).

In contrast to our study, astrocytes have previously been cultured in the presence of the growth factors FGF2 and EGF^5,15^, both of which are expressed in reactive astrocytes and allow the response to signaling pathways that are critical to neuronal fate choice^16–18^. To ask whether fundamental environmental differences affect neuronal reprogramming efficiency, we isolated astrocytes and cultured them in the continuous presence of FGF2 and EGF. By this growth factor treatment alone, a small percentage of βIII-tubulin^+^ cells appeared among control virus-infected cells (Fig. 3A and C), probably because FGF2 and EGF can confer stem cell-like properties on astrocytes, enabling them to differentiate into neurons in accordance with previous reports^19,20^. In addition, unlike in the absence of these growth factors (Fig. 2C,D), we found that even a single NeuroD1-virus infection together with FGF2 and EGF could effectively induce AtN conversion by 7 dpt (Fig. 3B,C). We then assessed whether subsequent FGF2 and EGF stimulation affect neuronal reprogramming from NR-astrocytes established in the absence of the growth factors. After 3 days’ stimulation with FGF2 and EGF, NeuroD1 expression was induced with 1 µg/ml of Dox in NR-astrocytes infected only once with NeuroD1-lentivirus. We found that FGF2 and EGF stimulation allowed NR-astrocytes exposed to a single NeuroD1 virus infection to be converted into neurons (Fig. 3D,E). In addition to FGF2 and EGF, LIF is expressed in reactive astrocytes and is known to affect their properties^21^. However, LIF stimulation did not improve the neuronal conversion efficiency of NR-astrocytes, although LIF stimulation in NR-astrocytes increased the expression of *Igf2*, which is known to be upregulated in reactive astrocytes (Supplemental Fig. 3A)^21^. Thus, these results indicate that environmental factors, especially those that confer stem cell-like properties on astrocytes, can contribute to efficient AtN conversion.

### Combinatorial expression of RFs enhances neuronal reprogramming

**Figure 4:**
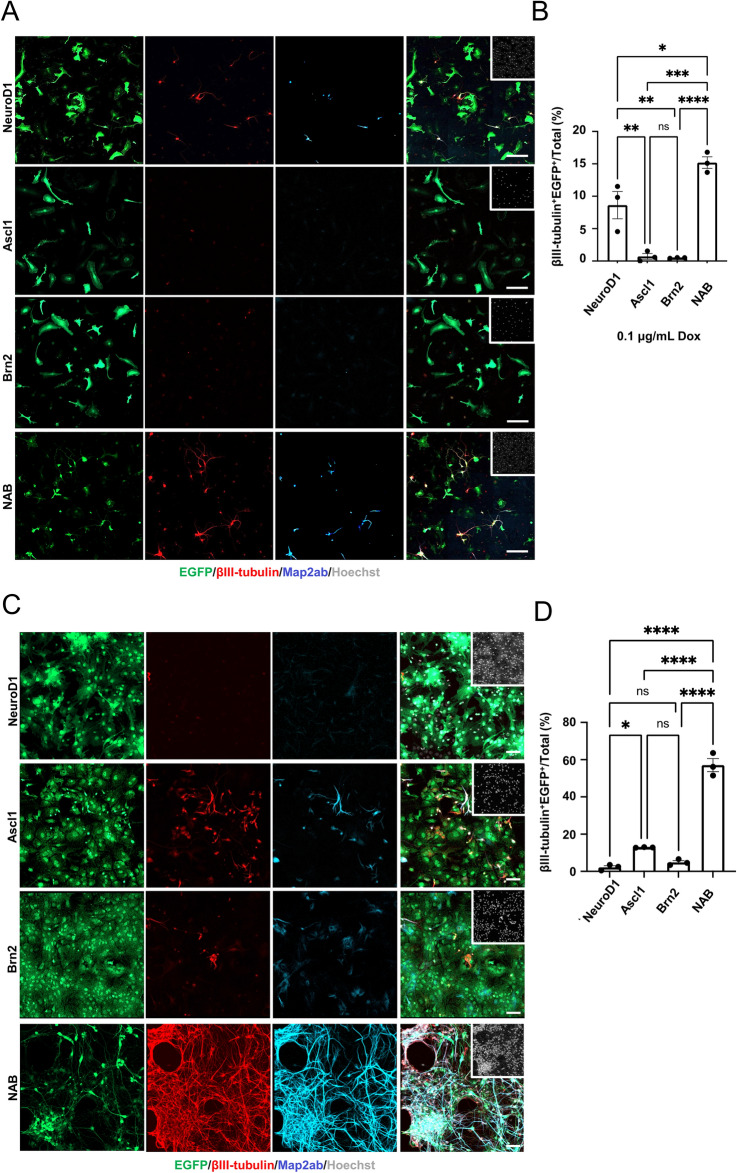
Optimal combinations of RFs increase neuronal reprogramming efficiency. (**A**) Representative images of staining for EGFP (green), βIII-tubulin (red), and Map2ab (cyan) in microglia infected with EGFP-and M2rtta-viruses together with NeuroD1-, Ascl1-, Brn2-, or NAB-viruses at 7 dpt. Dox, 0.1 μg/mL. Scale bars, 100 μm. (**B**) Quantification of the βIII-tubulin^+^ and EGFP^+^ cells in (A) (n = 3). ^*^p < 0.05 by unpaired Student’s t test. (**C**) Representative images of staining for EGFP (green), βIII-tubulin (red), and Map2ab (cyan) in NR-astrocytes (infected as in (**A**)) at 7 dpt. Dox, 1 μg/mL. Scale bars, 100 μm. (**D**) Quantification of the βIII-tubulin^+^ and EGFP ^+^ cells in (**C**) (n = 3). ^***^p < 0.0005 by unpaired Student’s t test.

Besides increasing the expression level of RFs and environmental factors, combinatorial expression of RFs is another strategy that should be considered to enhance neuronal reprogramming efficiency^3^. The neurogenic transcription factors Ascl1 and Brn2 have been shown to induce neuronal reprogramming from somatic cells, including astrocytes and microglia^10,22^. Therefore, we first expressed Ascl1 and Brn2 together with NeuroD1 (collectively, NAB) in microglia and found that this NAB combination augmented MtN conversion compared to NeuroD1 alone, even with a low dose (0.1 µg/mL) of Dox (Fig. 4A,B). We further observed that the efficiency of AtN conversion was dramatically increased by Dox (1 µg/mL)-induced NAB expression compared to NeuroD1 expression alone, in which AtN conversion was negligible (Fig. 4C,D). Furthermore, regardless of cell type, the percentages of neurons induced by the simultaneous infection with NAB-viruses were higher than the sum of those induced by each individual infection (Fig. 4A–D). These data suggest that the effective induction of neurons with the combinatorial expression of RFs did not result from the additive effect of the individual factors.

**Figure 5:**
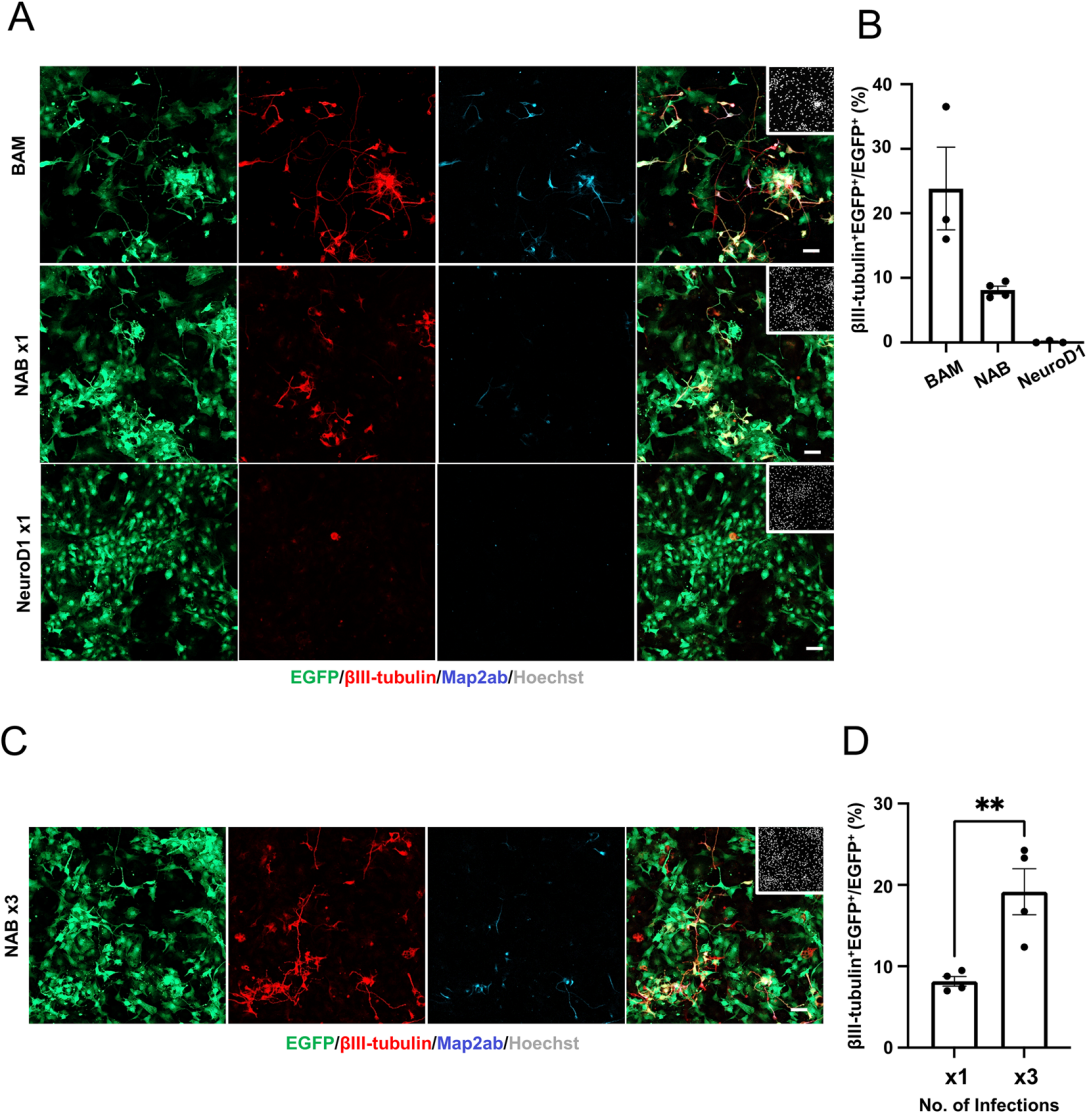
Combinatorial expression of RFs or multiple infection increases neuronal reprogramming efficiency in MEFs. (**A**) Representative images of staining for EGFP (green), βIII-tubulin (red), and Map2ab (cyan) in MEFs infected with EGFP- and M2rtta-viruses together with BAM-, NAB-, or NeuroD1-viruses at 7 dpt. Dox, 1 μg/mL. Scale bars, 100 μm. (**B**) Quantification of βIII-tubulin and EGFP^+^ cells in (**A**) (n = 3). (**C**) Representative images of staining for EGFP (green), βIII-tubulin (red), and Map2ab (cyan) in MEFs infected three times (× 3) with EGFP- and M2rtta-viruses together with NAB-viruses at 7 dpt. Dox, 1 μg/mL. Scale bar, 100 μm. (**D**) Quantification of βIII-tubulin and EGFP^+^ cells in NAB-transduced MEFs at 7 dpt (n = 3). Data for single NAB-transduced MEFs were obtained from quantification of βIII-tubulin^+^ and EGFP^+^ cells in (**C**) (n = 3). Data for the single simultaneous infection (× 1) with EGFP-, M2rtta-, and NAB-viruses are the same as those (NAB) in (B). ^**^p < 0.005 by unpaired Student’s t test.

We further examined whether combinatorial expression of RFs affects neuronal reprogramming from mouse embryonic fibroblasts (MEFs). MEFs were converted into neurons effectively by BAM (Brn2, Ascl1, and Myt1l) expression, in agreement with a previous report (Fig. 5A,B). Although MEFs infected with NeuroD1-virus alone were hardly converted into neurons, expression of NAB could efficiently convert MEFs into neurons (Fig. 5A,B). Moreover, repeated infections with NAB-viruses further promoted neuronal conversion from MEF (Fig. 5C,D). These results indicate that reprogramming efficiency can be positively modulated by combining optimal RFs even under conditions where reprogramming occurs inefficiently.

## Discussion

In this study, using NeuroD1 as a representative RF, we have shown that reprogramming efficiency is influenced by three factors: RF expression level, environmental factors, and the combination of RFs. In addition, we demonstrated that if the RF expression is sufficiently high, neurons can be induced with a single RF even in cells that are difficult to reprogram. In other words, this finding suggests that the key determinant of successful neuronal reprogramming among the three factors is the RF expression level.

We have previously revealed that in microglia, ectopically expressed NeuroD1 binds to closed chromatin with bivalent modifications, namely active (trimethylation of histone H3 at lysine 4 [H3K4me3]) and repressive (H3K27me3) marks, to induce the expression of neuronal genes^10^. In contrast to microglia, NR-astrocytes lack such bivalent signatures and exhibit a monovalent repressive modification (H3K27me3) around neuronal gene loci, in accordance with the low capacity of NeuroD1 to induce neuronal conversion of these cells^10^. However, another study demonstrated that NeuroD1 could occupy loci possessing the H3K27me3 modification to initiate neuronal programs in ES cells^23^. In the present study, we found that a relatively higher expression level of NeuroD1 induced by repeated virus infections could achieve neuronal reprogramming efficiently from NR-astrocytes. These findings suggest that while NeuroD1 preferentially binds to regions with bivalent modifications, an excess amount of NeuroD1 may increase the likelihood that it will also bind to regions with a monovalent repressive modification to initiate the neuronal program.

Recent researches have shown that the AtN conversion efficiency differs depending on the brain region in which the astrocytes reside. For example, astrocytes in the corpus callosum cannot be reprogrammed into neurons by expression of either NeuroD1 or the combination of Neurog2 and Nurr1, whereas astrocytes in the cortex can be^4,24^, implying that the particular environment in different brain regions dictates distinct astrocytic properties and consequently affects reprogramming potential. Astrocytes have been reported to acquire a variety of phenotypes and gene expression patterns in response to many pathological stimuli, such as stroke, neurodegenerative diseases, and aging^25^. We found in the present study that FGF2- and EGF-stimulated astrocytes are more likely to be converted into neurons than LIF-stimulated astrocytes, although all three of these factors are expressed in and regulate the behavior of reactive astrocytes in pathological conditions^21^. This result indicates that reprogramming efficiency from astrocytes may vary depending on brain pathologies as well as brain regions. Moreover, microglia have also recently been shown to manifest phenotypic heterogeneity across different regions and under neurological diseases in the brain^26,27^. Therefore, it is critical that neuronal reprogramming should be achieved by ensuring sufficient expression of RFs and, if necessary, examining their combinations to apply this technology to brain injury and disease therapy.

A recent study has reported that forced expression of NeuroD1 in microglia failed to convert them into neurons when a lentivirus harboring the CAG promoter, unlike ours, was used to express NeuroD1^28^. In that study, the exact amount of NeuroD1 expression was unknown since only relative quantification was performed. Based on our previous research, an efficient neuronal conversion of microglia can be induced with at least 3000 RPKM of NeuroD1 at 2 days after Dox treatment (1 μg/mL)^10,29^. In the present study, we have shown that microglia are hardly converted into neurons when NeuroD1 expression is low, but also indicated that increasing NeuroD1 expression dramatically enhances neuronal reprogramming from microglia. Therefore, failure of conversion into neurons does not necessarily mean that the original cells can never be converted to neurons by the expression of RFs, and it should be worthwhile to examine whether increased RF expression enables neuronal reprogramming from such cells.

Our findings provide insights into how RF expression levels affect neuronal reprogramming efficiency and ways to efficiently induce neurons from two glial and one non-glial cell types, i.e., microglia, astrocytes, and MEFs. Taking these observations into consideration, we believe that boosting reprogramming efficiency should offer therapeutic strategies for neurological conditions such as Alzheimer’s disease, spinal cord injury, and ischemia in the near future.

## Methods

### Isolation and culture of primary microglia and astrocytes

We prepared primary microglia and astrocytes from mouse at postnatal day 1 using a previously reported protocol^10^, with some modifications. We dissected cortexes of ICR mice after peeling of meninges to obtain microglia and astrocytes from glial cell mixtures. Dissected tissues were digested with papain (22.5 U/ml, Sigma) at 37 °C for 30 min and treated with DNase (200 U/ml, Sigma). After centrifugation (200 × *g*, 5 min), the cell pellet was suspended in alpha minimum essential medium (MEM) with 5% fetal bovine serum (FBS) and 0.6% glucose and filtered with a 40-μm cell strainer (BD Falcon). After centrifugation (200 × *g*, 5 min), the cell pellet was again suspended in alpha MEM and re-centrifuged (200 × *g*, 5 min). The cell pellets were resuspended in Dulbecco’s modified Eagle’s medium (DMEM)/Ham’s F-12 (Nacalai Tesque) containing 20% FBS, 1 mM L-sodium pyruvate, and MEM nonessential amino acids solution, and treated with GM-CSF (2.5 ng/mL; R&D Systems) to enhance microglial proliferation. This isolated glial mixture was incubated in T75 tissue culture flasks (BD Falcon) and the medium was changed every 2–3 days. Subsequently, we collected microglia by strong shaking for 1 h after 7–10 days in culture. The microglia were then plated onto an uncoated 35-mm culture dish and oligodendrocytes were removed by changing the medium 30 min after plating. We used the cells attached to the dish as primary cultured microglia and maintained them in DMEM/Ham’s F-12 containing 20% FBS, 1 mM L-sodium pyruvate, and MEM nonessential amino acids solution.

After collection of microglia, T75 flasks were treated with AraC (5 μM) for 2 days to remove proliferating cells. Cultures were shaken for 16 h, and then trypsin–EDTA solution was added to the flasks to obtain NR-astrocytes. Isolated NR-astrocytes were plated onto uncoated 35-mm culture dishes and maintained with DMEM/Ham’s F-12 containing 20% FBS.

To isolate FGF2- and EGF-stimulated astrocytes, the cell pellet obtained from dissected cortical tissues was cultured in T75 tissue culture flasks using DMEM/Ham’s F-12 containing 20% FBS, hEGF (10 ng/mL, Peprotech), hFGF2 (10 ng/mL, Peprotech), and B27. The medium was changed every 2 days. After 5–7 days of culture, trypsin–EDTA solution was added to the flask and the isolated cells were plated onto an uncoated 35-mm culture dish. FGF2 and EGF (10 ng/mL) or 50 ng/mL LIF were used to stimulate NR-astrocytes. All experimental procedures were approved by the Kyushu University Animal Experiment Committee, and the care and use of the animals were performed in accordance with institutional guidelines. In addition, all experimental protocols were approved by the Animal Care Committee of the Kyushu University. The study was carried out in compliance with the ARRIVE guidelines.

### Virus production

Lentiviruses were produced by transfecting HEK293T cells in a 10-cm dish with the constructs pCMV-VSV-G-RSV-Rev and pCAG-HIVgp using polyethylenimine. Since lot-to-lot variation in FBS preparations added to the culture medium critically influences the resultant viral tropism, we avoided using FBS for virus preparation^30^. After transfection, we cultured the cells with 5 mL of serum-free N2 medium (DMEM/F12 supplemented with insulin (25 μg/mL), apo-transferrin (100 μg/mL), progesterone (20 nM), putrescine (60 μM), and sodium selenite (30 nM)) for 2 days. The supernatant was collected and used for virus infection experiments after filtration through a 0.22 μm filter to remove cell debris. HEK293T cells kindly provided by Dr. Tetsuya Taga at Tokyo Medical and Dental University.

### Measurement of virus titer

The titers of recombinant viruses were checked using the Lenti-X GoStix Plus (Takara Bio) according to the manufacturer’s procedures. The measurement results of each titer (in IFU/mL) were are as follows: EGFP (5.5 × 10^5^); NeuroD1 (2.0 × 10^6^); Ascl1 (1.6 × 10^6^); and Brn2 (1.1 × 10^6^).

To measure virus titer from infected cells, we used an UltraRapid Lentiviral Glocal Titer Kit (System Biosciences) according to the manufacturer’s procedures. The results are as follows: NeuroD1 (4.9 × 10^6^ IFU/mL) and NAB (4.5 × 10^6^ IFU/mL).

### Induction of neuronal conversion

To induce neurons from glial cells, we used lentiviral vectors (derived from the Tet-O-FUW vector) in which gene expression of RFs and EGFP is controlled by the tetracycline operator. The vector backbone of the lentiviral plasmid expressing M2rtta is FUW. Plasmids used in this study are similar to those described in our previous report^10^. For cells to be infected efficiently with the lentivirus, the virus must be added as soon as possible after plating the microglia. Therefore, virus suspensions were added at the time of medium exchange 30 min after isolation of primary microglia, and infection was performed overnight. The medium was then replaced with a neuronal medium (Neurobasal Medium (Gibco) supplemented with B27 (Gibco), GlutaMAX (2 mM, Gibco), BDNF, GDNF, NT3 (10 ng/mL each, Peprotech), and penicillin/streptomycin/fungizone (Hyclone), and Dox induction was started for 7 days to convert microglia into neuronal cells. Dox was added only once to the medium to activate RF expression. The medium was changed every 2–3 days for the duration of the culture period.

For conversion into neuronal cells from astrocytes, the virus suspension was added at the time the cells were seeded, and infection was performed overnight. The medium was replaced with neuronal medium the next day, and Dox induction was started for 7 days to convert astrocytes into neuronal cells.

For sequential viral infections, a second infection was performed 8 h after the first infection was completed, and a third infection 8 h after the second, and overnight virus infection and medium replacement were repeated. To convert glial cells into neuronal cells, the medium was replaced with neuronal medium containing Dox after the final infection and the cells were cultured for 7 days. The medium was changed every 2–3 days for the duration of the culture period.

### MEF isolation and culture, and induction of neuronal reprogramming

MEFs were isolated from E14 mouse embryo bodies. The bodies, from which the head, organs, and tail had been removed, were dispersed using papain (22.5 U/mL, Sigma) at 37 °C for 30 min and treatment DNase (200 U/mL, Sigma). After centrifugation (200 × *g*, 5 min), the cell pellet was suspended in DMEM containing 4.5 g/L glucose (Nacalai) with 10% FBS and filtered with a 40-μm cell strainer (BD Falcon). After centrifugation (200 × *g*, 5 min), the cell pellets were resuspended in DMEM containing 4.5 g/L glucose with 10% FBS. MEFs were then plated onto an uncoated 100-mm culture dish. The medium was changed the following day. After 2 days of culture, we passaged the MEFs into a new culture dish and began to induce neuronal reprogramming with virus infection.

To induce neurons from MEFs, we used lentiviral vectors in which gene expression is controlled by the tetracycline operator, as described in the main text. Virus suspensions were added 3 h after plating the MEFs, and infection was performed overnight. The medium was then replaced with a neuronal medium (Neurobasal Medium (Gibco) supplemented with B27 (Gibco), GlutaMAX (2 mM, Gibco), BDNF, GDNF, NT3 (10 ng/ml each, Peprotech), and penicillin/streptomycin/fungizone (Hyclone)), and DOX induction was started for 7 days to convert MEFs into neuronal cells. DOX was added only once to the medium to activate RF expression. The medium was changed every 2–3 days for the duration of the culture period.

For sequential viral infections, a second infection was performed 8 h after the first infection was completed, and overnight virus infection and medium replacement were repeated. To convert glial cells into neuronal cells, the medium was replaced with neuronal medium containing Dox after the final infection and the cells were cultured for 7 days. The medium was changed every 2–3 days for the duration of the culture period.

### Immunochemistry

Cells were fixed in 4% paraformaldehyde for 10 min and blocked for 1 h at room temperature (RT) with blocking buffer (5% FBS and 0.3% Triton X-100). After blocking, the cells were incubated with the following primary antibodies for 2 h at RT: anti-βIII-tubulin (1:500, Covance), anti-Map2ab (1:500, Sigma), anti-GFP (1:500, Aves), anti-NeuroD1 (1:200, Abcam), anti-Tmem119 (1:500, Abcam), anti-CD68 (1:500, Bio-Rad), and anti-Iba1 (1:500, Abcam). Stained cells were visualized with a fluorescence microscope (Axiovert 200 M, Zeiss) and a confocal microscope (LSM800, Zeiss). Fluorescence intensity of the cell soma was quantified using LAS AF (Leica) or ZEN (Zeiss).

### Real-time qRT-PCR

Total RNA was isolated using an RNeasy Micro Kit (QIAGEN) according to the supplier’s protocol. RNA quality was checked with a spectrophotometer. Reverse transcription reactions were performed using a SuperScript VILO cDNA Synthesis Kit (Life Technologies) following the supplier’s protocol. qRT-PCR was performed with SYBR green fluorescent dye using Step One Plus (Applied Biosystems) and Mx3000 (Stratagene). GAPDH was used as an endogenous control to normalize samples. The PCR primers used in this study were listed as follows.

Total mNeuroD1:

Fw: AAGCCACGGATCAATCTTCTC.

Rv: CGTGAAAGATGGCATTAAGCTG.

Exogenous NeuroD1:

Fw: CAGCTTAATGCCATCTTTCACG.

Rv: CATAGCGTAAAAGGAGCAACA.

Igf2:

Fw: GTACTTCCGGACGACTTCCC.

Rv: TCAGGGGACGATGACGTTTG.

### Statistical analysis

Data were analyzed using Prism 9 ver.9.1.2. Unpaired Student’s t tests were used to calculate the p value for pairwise comparisons. For multiple comparisons, p values were calculated using one-way ANOVA with the Tukey post hoc test. Data represent mean ± SEM. We considered probabilities of *p* < 0.05 to be significant.

## Supplementary Information

Supplementary Information.

## Data Availability

Any data generated form these studies is available from the corresponding author upon reasonable request.
