# Supplementary material for: Expression level of the reprogramming factor NeuroD1 is critical for neuronal conversion efficiency from different cell types
Source: Sci Rep. 2022 Oct 26;12:17980. doi: 10.1038/s41598-022-22802-z (PMC9606360; doi:10.1038/s41598-022-22802-z)
Supplement: Supplementary file 1 — Supplementary Information. [file 41598_2022_22802_MOESM1_ESM.docx]

**Matsuda-Ito, et al. “Expression level of the reprogramming factor NeuroD1 is critical for neuronal conversion efficiency from different cell types”**

**Supplemental Figures**


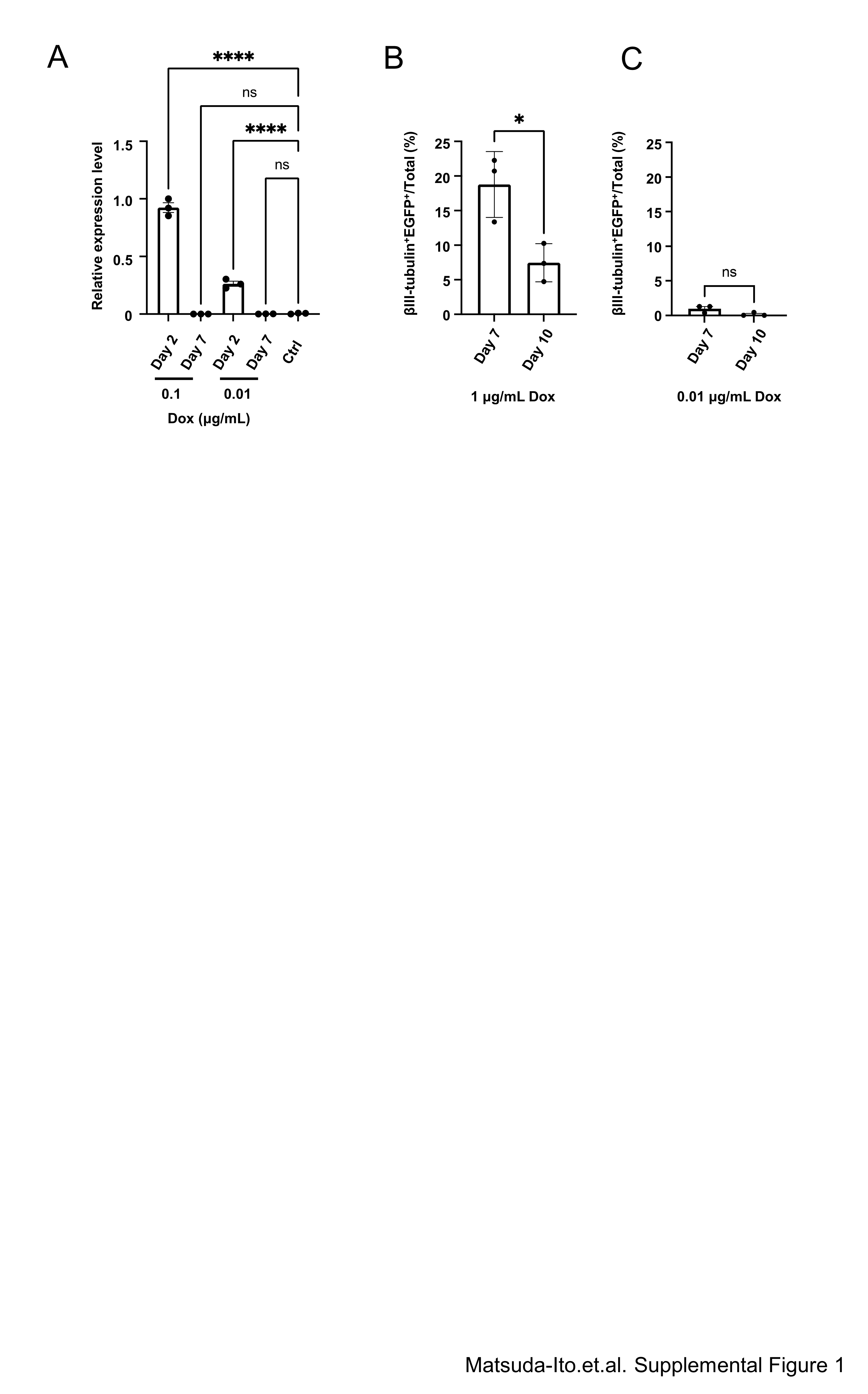


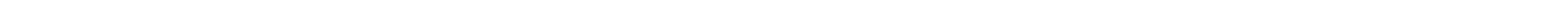


**Supplemental Figure 1 Transient NeuroD1 expression induces neuronal reprogramming from microglia**

(A) qRT-PCR analysis of exogenous NeuroD1 levels in EGFP-, M2rtta-, and NeuroD1-virus-infected microglia at 2 dpt and 7 dpt under the indicated Dox concentrations. Ctrl indicates EGFP- and M2rtta-virus-infected microglia. (n = 3 biological replicates). ****p < 0.0001 by ANOVA with Tukey *post hoc* tests. ns means not significant (p > 0.05).

(B) Quantification of the βIII-tubulin and EGFP^+^ cells at 7 dpt and 10 dpt (n = 3). Data for ND1-transduced microglia at 7 dpt were obtained from quantification of βIII-tubulin and EGFP^+^ cells in Fig. 1D. Dox, 1 μg/mL. *p < 0.05 by unpaired Student’s t test.

(C) Quantification of the βIII-tubulin and EGFP^+^ cells at 7 dpt and 10 dpt (n = 3). Data for ND1-transduced microglia at 7 dpt were obtained from quantification of βIII-tubulin and EGFP^+^ cells in Fig. 1D. Dox, 0.01 μg/mL. ns means not significant (p > 0.05).


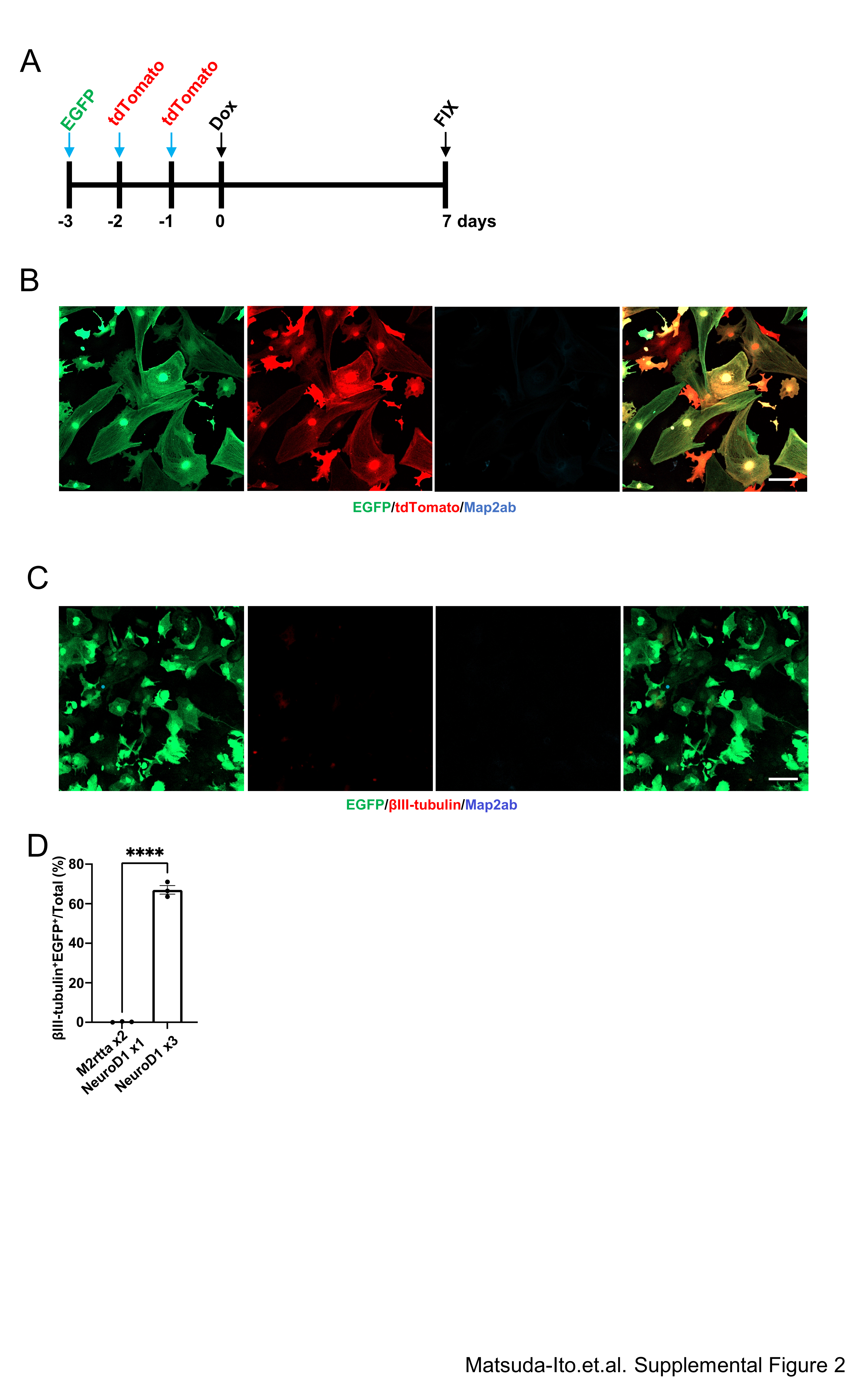


**Supplemental Figure 2 Multiple infections are observed in a single cell**

(A) Scheme of multiple infections of NR-astrocytes with EGFP- and tdTomato-viruses.

(B) Representative images of staining for EGFP (green), tdTomato (red), and Map2ab (cyan) in multiply virus-infected NR-astrocytes at 7 dpt. Dox, 1 μg/mL. Scale bar, 100 μm.

(C) Representative images of staining for EGFP (green), βIII-tubulin (red), and Map2ab (cyan) in NeuroD1-transduced astrocytes at 7 dpt. NR-astrocytes were first infected twice with two viruses (M2rtta- and EGFP-viruses; indicated in (D) as M2rtta ×2) and then once with three viruses (M2rtta-, EGFP- and NeuroD1-viruses; indicated in (D) as NeuroD1 ×1). Dox, 1 μg/mL. Scale bar, 100 μm.

(D) Quantification of the βIII-tubulin and EGFP^+^ cells in (C) and in NR-astrocytes infected three times with NeuroD1-containing viruses (NeuroD1 ×3). Data for NeuroD1 ×3 are the same as those (NeuroD1 ×3) in Figure 2F. (n = 3). ****p < 0.0001 by unpaired Student’s t test.


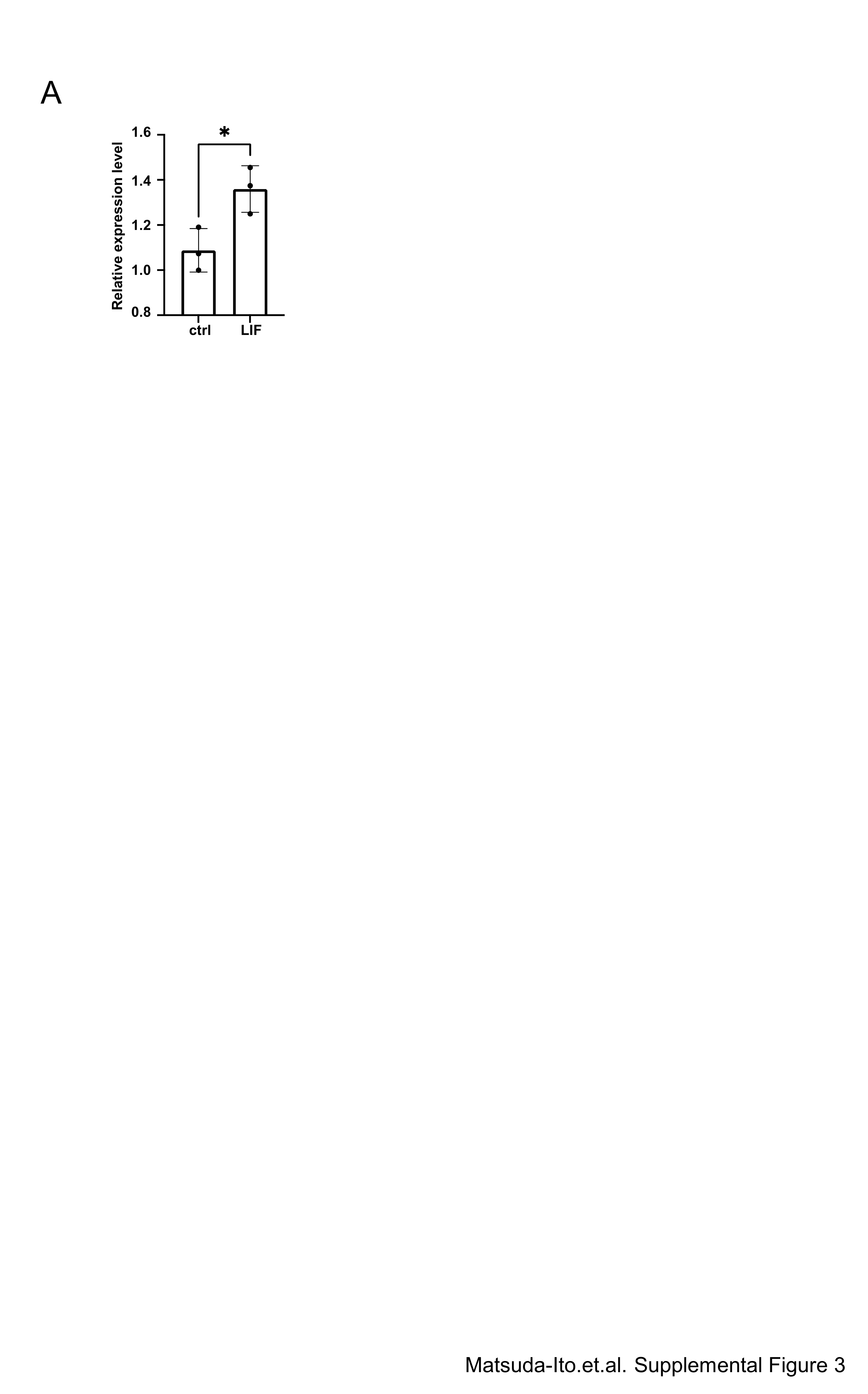


**Supplemental Figure 3 LIF-stimulated astrocytes express *Igf2***

(A) qRT-PCR analysis of total *Igf2* mRNA levels in LIF-stimulated astrocytes after 1 day (n = 3 biological replicates). *p < 0.05 by unpaired Student’s t test.
